# Longevity and Efficiency

**Published:** 1903-11

**Authors:** William W. Marvel

**Affiliations:** Fall River, Mass.


### LONGEVITY AND EFFICIENCY.[1]

BY WILLIAM W. MARVEL, D.M.D., FALL RIVER, MASS.

FELLOW-MEMBERS OF THE HARVARD DENTAL ALUMNI ASSOCIATION,—It is not enough to be contented with day-to-day freedom from discomfort or pain, but we ought, rather, to look towards longevity as a means of rounding out our professional career in doing as large an amount of good to as many of our fellows as possible. The question whether "life is worth living" has been decided by a majority far too great to admit of any doubt upon the subject, and the voices of those who would fain reply in the negative are drowned amid the chorus of assent. Longevity, indeed, has come to be regarded as one of the grand prizes of human existence, and reason has again and again suggested the inquiry: whether skill can increase the chances of acquiring it, and can make old age, when granted, as comfortable and happy as any other stage of our existence.

Now, how many dental practitioners, restricted to four walls, with apparently nothing but conditions adverse to perfect health, live long and still keep up with the progress of the times in their chosen profession? I am sure this is a very practical question with every man who has been in practice even a few years, especially if he has become fairly busy. So rapidly have been the new developments in the dental world, not simply in fuller knowledge in old fields, but in the broader knowledge taking in new subjects, that for one to attempt to keep pace with it all would require one's entire time for study and investigation. It is perhaps the feeling of almost hopeless desperation at one's inability to accomplish this and still do the urgent crowding work with which every day is filled. The impossibility of doing all that one would like has forced many to say to themselves, having been left behind by the too rapid pace, "We cannot cover the whole domain of dental progress, and will select one department." True, many of us are instinctively drawn by particular aptitude to the special sphere of oral surgery, orthodontia, or porcelain work, and by following one branch zealously and keeping abreast of the times, or even a step in advance,

---

[1] Read before the Harvard Dental Alumni Association, Boston, Mass., Monday, June 22, 1903.

may become identified with one particular branch of our profession. This may be a solution of the question, so far as they are concerned. But it is evident that in a suburban or country practice we must be fairly well up in the whole field, and must devote a good portion of our time to study and investigation. We are apt to hold ourselves too closely to the bare routine, confining our practice to plain amalgam, cement, or gold fillings, without thinking about what may be accomplished for our own betterment and that of our patient by porcelain work or burnished fillings. We all hear about the preservative property of tin, and have seen clinicians insert fillings of that material; have become enthusiastic, perhaps, in speaking of it to our fellows; but who of us put tin to the practice, and see for ourselves whether or not it is a good thing? We may attend a dental meeting, go to our office primed for increasing our scope, and then, when a case presents itself, say, "Oh, I have not time to bother with it. I think I will put in amalgam; I have used it successfully for twenty years, and I might make a botch of something else." Can we ever hope to progress with such a weak idea of our own ability?

Now, we cannot expect to have a healthy mind without a healthy growth of new ideas, and no matter how healthy our mind is, we cannot put these ideas to use without a healthy body and steady nerves. As old Democritus aptly said, "The force of the understanding increases with the health of the body. When the body labors under disease, the mind is incapacitated for thinking;" and the president of our own University has said, "To attain success and length of service in any of the professions, a vigorous body is essential. All professional biography teaches that to win distinction in sedentary, in-door occupations, which task the brain and nervous system, extraordinary toughness of the body must accompany extraordinary mental powers." Probably no single calling in life demands more of this bodily ruggedness than our own. Constantly standing, often in a cramped position, operating over a living, sensitive being, necessary at times to inflict pain, to endure offensive breaths, to inhale anything but pure air in a 6 x 10 room; through all this, all day long, day after day, we are required to have a steady hand and a cheerful countenance. Unfortunately, too few of us have a system connected with our daily life,—regular time for rising, simple yet wholesome breakfast, regular time for beginning and ending work, and regular exercise.

How much better we would be if a walk of a mile or so after breakfast were indulged in! We could meet our first patient in the morning with a glow on our face and a cheerful word impossible to the dyspeptic or overworked man.

When we pick out an office we should choose it as we would our sleeping-apartments: plenty of fresh air, even in winter; better have the rest of the house overheated, for the sake of having a window open in the operating-room. As to exercise, the methods are too numerous to mention them all. Nothing can surpass the out-door exercise waiting for us to enjoy: membership in a tennis or golf club; daily horseback riding in the early morning or evening, or the frequent walks connected with automobiling. In winter we need not neglect our exercise on account of the weather, with so many gymnasia handy. However, exercise and fresh air are not the only exponents of the healthy body. We must select our food. The simple fare of the laborer is a blessing to him, but while the luxurious diet of the wealthy gourmand affords temporary pleasure, it hides beneath the palate, in tempting viands, a weapon which strikes deep into his vitals, and brings the culprit to nature's judgment bar for punishment. We must build our bodies as well as build our practice. The attitude of mind with multitudes, in reference to the matter of eating, is, "Let us eat, drink, and be merry," forgetting the thought which naturally follows, "For to-morrow we die." "A short life, and a merry one," is a popular modern adage, which contains in itself the confession or the recognition of the relation between a merry life, or, rather, a life of self-gratification and brevity of years. If one would live long, he must eat well; he must live high in the true sense; that is, he must live in harmony with high principle, in accordance with divine order; he must recognize the fact that the stomach may become likewise the fountain-head of disease of every kind and in every tissue.

It seems like "carrying coals to Newcastle" to say to men affiliated with the healing art, that a man can do his best work only when he is in good physical condition, and that good physical condition comes not with neglect of it, but with the cultivation of it.

It is unwise devotion even to a good cause that persistently violates the laws of health in reserving for one's self not time enough for sleep or for physical, social, intellectual, and moral exercise outside the immediate duties of one's calling. The public is exacting, our own ambition is more exacting, but the laws of health are even still more exacting. It would seem that, with the best system possible for securing the dentist's rights to himself, it is almost impossible, as one goes along, to keep even with the world. Where the debt of time for rest and recreation is, in spite of himself, constantly accumulating, there is no other remedy but to exact payment by a vacation (annually) long enough to liquidate the account for lost nights and loss of control over our nerves. This is needed for the sake of health, but the infringement on the man's time needed for self-improvement, for study in his profession, for general culture, demands a like liquidation. Whether this may be best balanced by time each year set apart for this purpose, or whether the obligation may be allowed to accumulate on the "ten-year life insurance dividend period basis," it must be met, if one is to keep a high place in his profession.

Much could be said about the proper way to live our daily life, our attention to our eyes, our posture while operating, and scores of other points, but I have in this brief paper attempted to show that in this age of high pressure and rapid progress the dentist, both as a man and as a machine, needs more time than is usually taken for repair and recuperation. By various devices alluded to he may, as he goes on, reserve some time for health and culture, but, apart from this, for the purpose, more time must be taken and more opportunity sought regularly. It may seem to some a losing investment at the time, but is more likely to prove profitable, whther as regards the greater pleasure, the larger usefulness, or the longer lease of life.

